# SLM-processed MoS_2_/Mo_2_S_3_ nanocomposite for energy conversion/storage applications

**DOI:** 10.1038/s41598-022-08921-7

**Published:** 2022-03-23

**Authors:** Navid Alinejadian, Sayed Habib Kazemi, Inger Odnevall

**Affiliations:** 1 Department of Mechanical and Industrial Engineering, Tallinn University of Technology, 19086 Tallinn, Estonia; 2 KTH Royal Institute of Technology, Division of Surface and Corrosion Science, Department of Chemistry, School of Engineering Sciences in Chemistry, Biotechnology and Health, Drottning Kristinas väg 51, 100-44 Stockholm, Sweden; 3 Department of Chemistry, Institute for Advanced Studies in Basic Sciences, Zanjan, 45137-66731 Iran; 4 AIMES-Center for the Advancement of Integrated Medical and Engineering Sciences, Karolinska Institutet, KTH Royal Institute of Technology, Stockholm, Sweden; 5 Department of Neuroscience, Karolinska Institutet, 171 77 Stockholm, Sweden

**Keywords:** Materials for energy and catalysis, Composites, Energy storage

## Abstract

MoS_2_-based nanocomposites have been widely processed by a variety of conventional and 3D printing techniques. In this study, selective laser melting (SLM) has for the first time successfully been employed to tune the crystallographic structure of bulk MoS_2_ to a 2H/1T phase and to distribute Mo_2_S_3_ nanoparticles in-situ in MoS_2_/Mo_2_S_3_ nanocomposites used in electrochemical energy conversion/storage systems (EECSS). The remarkable results promote further research on and elucidate the applicability of laser-based powder bed processing of 2D nanomaterials for a wide range of functional structures within, e.g., EECSS, aerospace, and possibly high-temperature solid-state EECSS even in space.

## Introduction

Na-S energy conversion/storage devices have earlier been demonstrated as possible alternatives to mitigate thermal decomposition, short circuit, heat, fire, or explosion of Li-ion electrodes^1,2^. Recently has a high thermal stable hexagonal boron nitride (h-BN) been introduced as a promising anode material, though its low electrical conductivity may limit its electrochemical performance^1^. Molybdenum disulfide (MoS_2_) belongs to a class of advanced next-generation materials called Transition Metal Dichalcogenides (TMDs) with a direct bandgap of ~ 1.8 eV and a melting point of 1185 ºC in nonoxidizing environments^3^. MoS_2,_ with strong covalent S–Mo–S bonds and weak inter-layer van der Waals forces^4^, and its nanocomposites dominate electroactive layered structures due to their transitional structure from semiconductor 2H to metallic 1T, which is favorable in electrochemical applications^5,6^. Similar to Li^+^, the accommodation of Na^+^ between 1T-MoS_2_ results in considerable capacitance and interesting electrochemical properties^7^.

MoS_2_-based nanocomposites have been widely studied using a variety of conventional methods^8–12^ which have different technical drawbacks such as complex expensive pre-processing (exfoliation, preparation, and large-scale production), processing (deposition or lack of precise morphology control), and complementary post-processing stages (thermal, acid treatment, and limitation of geometric and specific surface area)^6,13^. Despite the mitigation of existing drawbacks, scientists have enhanced processing possibilities which have led to novel 3D printing techniques^14–20^. The use of these techniques is still limited due to drawbacks such as restricted resolution, weak mechanical properties, low printing speed, or high cost of equipment^6,21–23^.

Laser-based thinning can effectively exfoliate MoS_2_ in a controlled manner^24^ to enable green fabrication of 3D shapes from 2D nanomaterials^25,26^, and improve the charge capacitance properties of Mo_2_S_3_ nanostructures^27^. The thinning mechanism of MoS_2_, with a high laser absorption coefficient due to the weak van der Waals forces between poorly coupled MoS_2_ layers, can be attributed to localized temperature-dependent sublimation of upper layers of bulk 2H-MoS_2_ lattice, arising through the absorption of high-energy laser^24,25,28^. At the next steps, laser irradiation on thinned 2H-MoS_2_ can lead to further photoinduced exfoliation to form few-layered nanosheets^25^. On the other hand, the more laser reflection due to the high reflection ratio of Mo powder^28^, the more thinning of 2H-MoS_2_ existing nearby Mo powders^25^.

2H-MoS_2_ is the thermodynamically stable trigonal phase consisting of prismatic coordination of Mo atoms by six surrounding sulfur atoms, whereas metallic 1T-MoS_2_, metastable octahedral coordination of Mo atoms in MoS_2_, can be a result of the first-order phase transition due to the change in the density of states^29^. It has already been shown that the exfoliated form consists of a 1T-MoS_2_ phase with lattice distortions according to the exfoliation technique. The annealing of metastable 1T-MoS_2_ at temperatures lower than 300 ºC can lead to restacking of the layers and restoration of 2H-MoS_2_^7,29,30^. Hence, generally, integrated hybrid 1T/2H-MoS_2_ exists in which the 2H phase can stabilize the metastable 1T phase, avoiding restocking and restoration^30^. It has though been proven that 2H-MoS_2_ at temperatures higher than 600 ºC can be transformed into 1T-MoS_2_ via nucleation of an intermediate and subsequently 2H/1T boundaries migration over time^31^.

Accordingly, high-energy laser processing of MoS_2_ can impose a highly localized temperature resulting in the thinning of MoS_2_ as well as the creation of many α-phase nucleation sites for 2H to 1T phase transformation^25,26,31^. Mo_2_S_3_ nanostructures can due to surface phase transformation also be formed through thermal annealing or sputtering of MoS_2_ in an Ar-isolated atmosphere and at high temperatures above 1300 ºC^25^. Imposing sulfur vacancies into the structure of MoS_2_ in the exposure of high-energy laser beam in the presence of melted Mo at a high temperature can also result in the formation of Mo_2_S_3_ nanostructures at the edges of laser-thinned 1T-MoS2 and sulfur vacancies^25,26^. Consequently, the rapid cooling rate of the SLM process as well as the presentation of Mo_2_S_3_ nanostructures can significantly prevent the restoration of 1T/2H-MoS_2_.

Compared to other 3D printing methods, SLM relies on versatile high-energy Nd:YAG laser equipment which enables printing of parts of improved mechanical properties and better resolution. Since the SLM technique can overcome many of the demerits of both conventional and other 3D printing techniques^6,21,23^, this technology can be used for rapid prototyping of future energy storage materials^6,32^. Despite the specific characteristics of MoS_2_ in electrochemical applications, the weak van der Waals forces make direct SLM processing of MoS_2_ structures difficult. The addition of Mo powder, as a metallic additive, has been shown to both facilitate the printability of MoS_2_ based structures and result in the formation of stable electrocatalytic Mo_2_S_3_ nanostructures via Mo–Mo zigzag chains, which enhance the electron transfer within and between the S–Mo–S layers^27^.

This study reveals the extraordinary influence of the SLM technology not only on the crystallographic phase transformation of MoS_2_ from bulk 2H to metallic 1T through one-step laser-based exfoliation but also on the simultaneous formation and uniform distribution of Mo_2_S_3_ nanoparticles in the nanocomposite structure. The unprecedented electroactivity of the one-step SLM-processed MoS_2_/Mo_2_S_3_ nanocomposite (SLM-Mo_(x)_S_(x+1)_) is demonstrated, paving the way for the fabrication of the next generation EECSS. Our recent findings demonstrate that the SLM technique is able to directly utilize raw materials, exfoliate MoS_2_ in-situ during fabrication of intricate parts, such as electroactive components of EECSS, and directly deliver parts of different functionalities without any special additives or binders.

## Results

**Figure 1:**
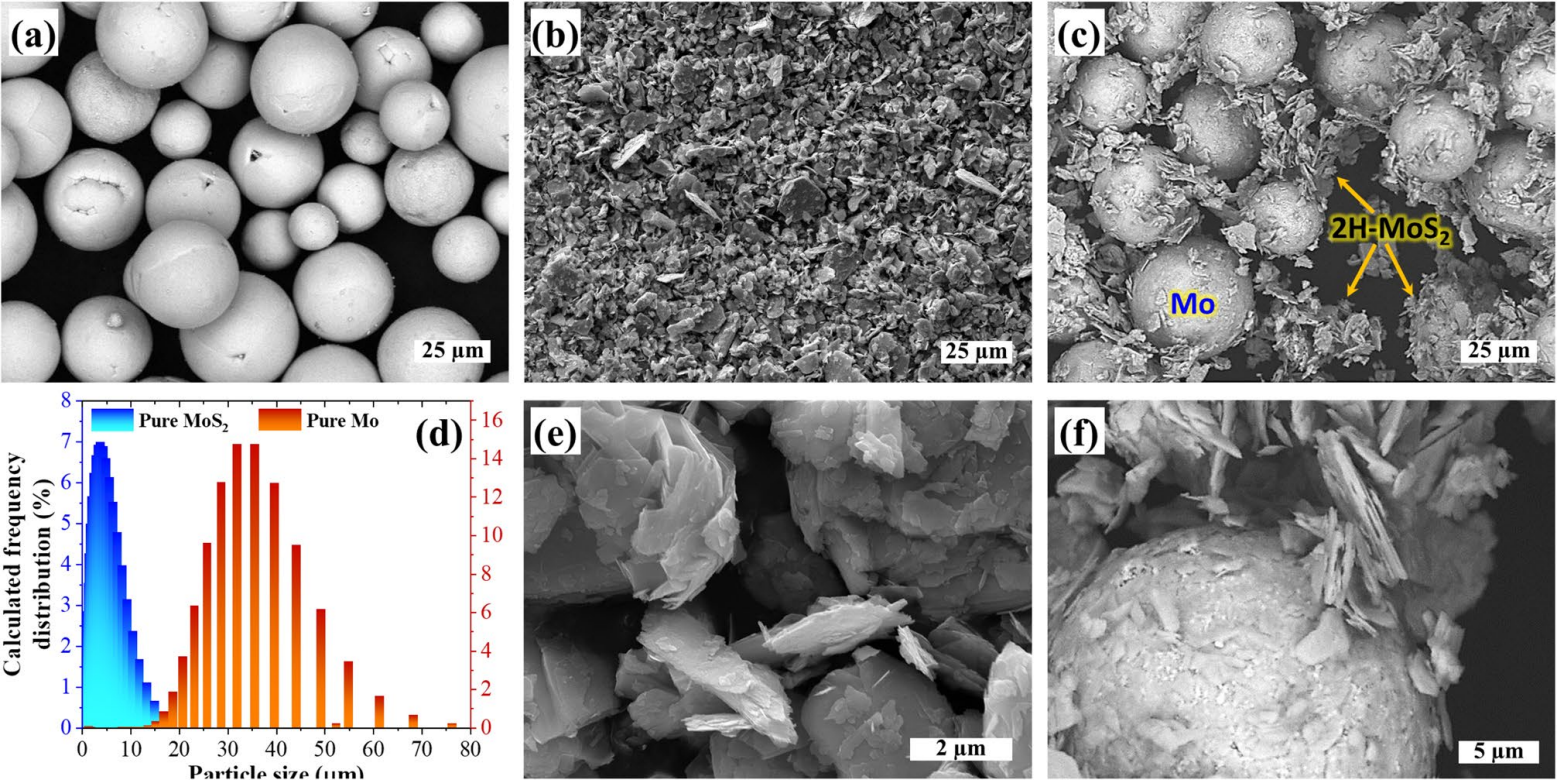
Scanning electron microscopy images of **(a)** pure Mo powder, **(b)** layered MoS_2_ powder, and **(c)** Mo-MoS_2_ powder mixture feedstock. **(d)** Particle size distribution plot of Mo and MoS_2_ powders based on high-resolution scanning electron microscopy images of **(e)** layered MoS_2_ powder and **(f)** the Mo-MoS_2_ mixture feedstock.

The spherical morphology of Mo and irregularly shaped MoS_2_ particles can be ascertained from Fig. 1a,b,e, showing an average size (d_50_) of 25–35 µm and 2.5–7.5 µm, respectively (Fig. 1d). Aside from slightly crushed pure MoS_2_, its lamellar morphology was retained even after 6 h of mixing with pure Mo (Fig. 1c,f), enhancing the packing density^33^ and the mean laser absorptivity^25,28^ of the Mo-MoS_2_ mixture feedstock.

**Figure 2:**
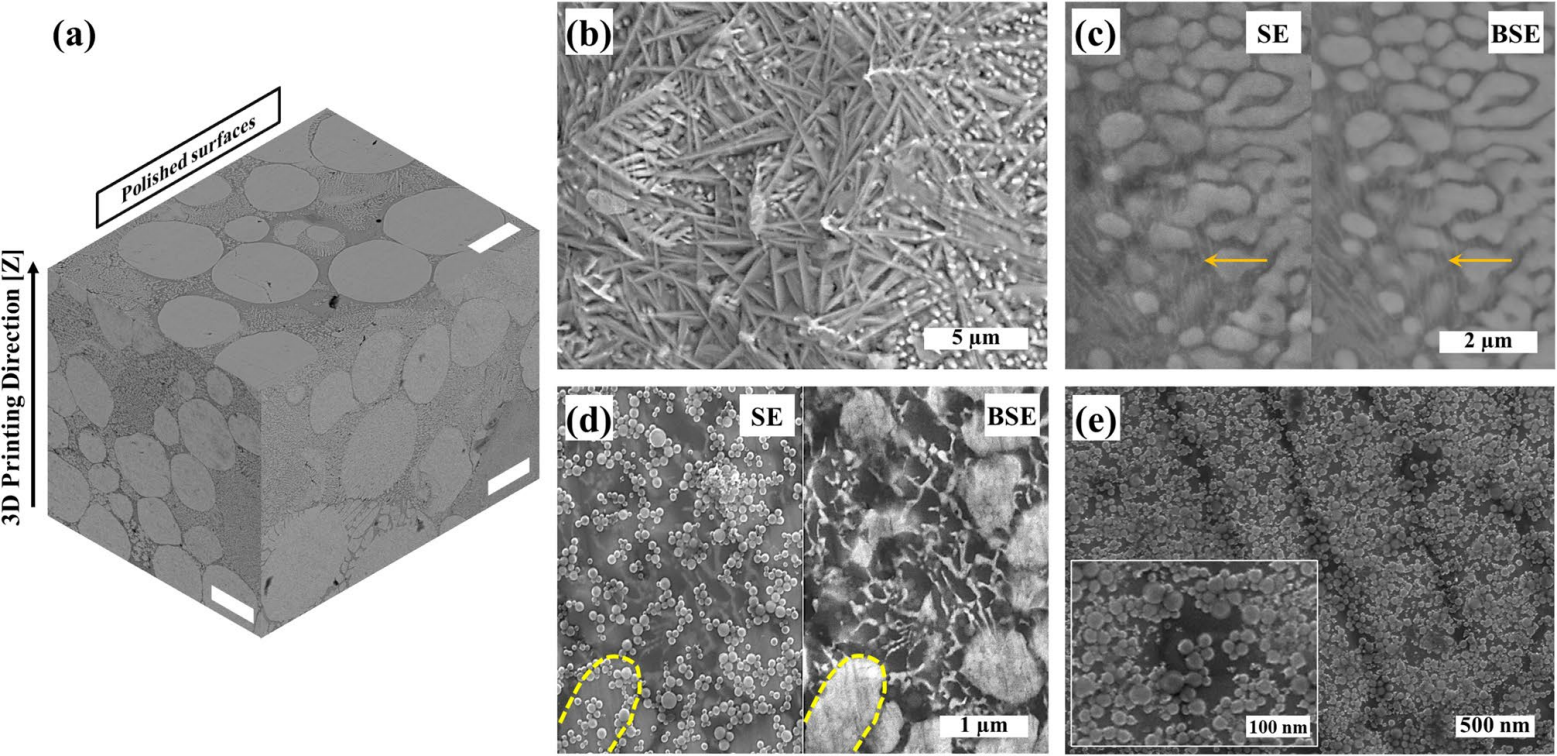
SEM micrographs of **(a)** polished surfaces of SLM-Mo_(x)_S_(x+1)_ (scale bar = 25 µm), **(b)** unpolished top-surface including laser-assisted exfoliated 1T-MoS2 nanosheets and Mo_2_S_3_ nanoparticles, **(c)** high magnification secondary and backscattered electron micrographs acquired from the polished cross-section in which the arrows indicate thin transparent 1T-MoS_2_ layers alongside coalesced Mo_2_S_3_ nanoparticles, **(d)** HR-SEM micrograph of the polished surface of SLM-Mo_(x)_S_(x+1)_ in which the yellow dashed lines indicate coalescence zones of Mo_2_S_3_ nanoparticles, and **(e)** high magnification HR-SEM micrograph of a cross-section showing Mo_2_S_3_ nanoparticles within the structure of the nanocomposite.

Illustrated in Fig. 6e (experimental), three types of defects were observed in the laser single scan (LSS) of powder bed; (i) lack of fusion (LOF) defects which were intensified by increased scan speed and reduced laser power, (ii) balling defects due to the insufficient imposed energy density either at high scan speeds or low laser powers, and (iii) over melting of the powder bed at high energy densities due to both high laser power and low scan speed^28^. The 3D SEM micrograph from different polished sections of SLM-Mo_(x)_S_(x+1)_ indicated a similar microstructure which can be ascribed to the optimized LSS (laser power of 62.5 W and a laser scan speed of 85 mm s^−1^) resulting in a relatively stable melt-pool with a nearly constant thermal gradient during the SLM process (Fig. 2a)^34,35^. Scanning electron micrographs (SEM) illustrate the exfoliation of bulk 2H-MoS_2_ into thin 1T-MoS_2_ nanosheets and the distribution of Mo_2_S_3_ nanoparticles in the matrix (Fig. 2b). Both the back-scattered (BSE) and the secondary-electron (SE) micrographs elucidate the presence of 1T-MoS_2_ layers of low density (Fig. 2c,d). Indicating identical zones, the arrows in Fig. 2c and the dashed lines in Fig. 2d, confirm the presence of Mo_2_S_3_ nanoparticles coalesced at the lower parts- and separated at the top surface of the nanocomposite, delimited by a transparent 1T-MoS_2_ layer. The coalescence is attributed to partial remelting of the subsequent layers during the layer-wise SLM process. HR-SEM micrographs (Fig. 2e) reveal a uniform distribution of Mo_2_S_3_ nanoparticles sized between 25 and 50 nm. The spherical particle morphology is attributed to the high surface tension between MoS_2_ and Mo_2_S_3_ due to varying Mo/S ratios and the tendency of resolidified nanostructures to reduce their surface energies^25^.

**Figure 3:**
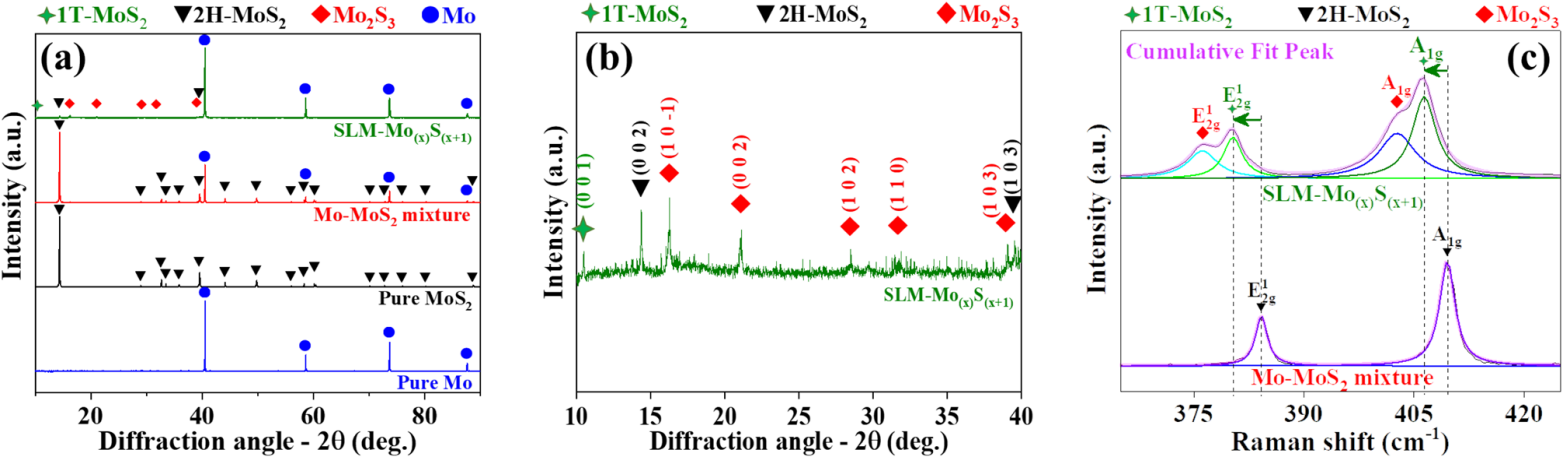
**(a)** XRD patterns of the pure Mo powder, MoS_2_ powder, Mo-MoS_2_ powder mixture, and SLM-Mo_(x)_S_(x+1)_ samples, **(b)** magnified XRD peaks between 10 ≤ 2ϴ ≤ 40 showing the presence of 1T-MoS_2_, 2H-MoS_2_, and Mo_2_S_3_ phases in the nanocomposite and **(c)** Raman spectra with the deconvoluted profiles of 1T-MoS_2_, 2H-MoS_2_, and Mo_2_S_3_ nanoparticles showing the shift in their position.

The X-ray diffraction (XRD) patterns verified the purity of Mo and MoS_2_ both before and after the mixing process (Fig. 3a). The low-intensity XRD peaks confirm the presence of Mo_2_S_3_ along with pure 1T/2H-MoS_2_ crystallographic planes (Fig. 3b). Both the exfoliation and the phase transformation of 2H-MoS_2_ resulted in diffraction peaks related to the 001 plane of 1T-MoS_2_ (2θ = 10.495º)^7^. Illustrated in Fig. 3c, the broadening and blue shift of the ${\mathrm{E}}_{2\mathrm{g}}^{1}$ and ${\mathrm{A}}_{1\mathrm{g}}$ Raman peaks from 384 cm^−1^ (${\mathrm{E}}_{2\mathrm{g}}^{1}$) and 409 cm^−1^ to 380 cm^−1^ and 406 cm^−1^, respectively, confirm the Nd:YAG laser-assisted thinning and transformation of 2H-MoS_2_ to a few-layered 1T-MoS_2_ at the temperature elevated above 650º K^25,26^. Besides, the existence of Mo_2_S_3_ nanoparticles was verified by the appearance of two shoulders associated with ${\mathrm{E}}_{2\mathrm{g}}^{1}$ and ${\mathrm{A}}_{1\mathrm{g}}$ Raman peaks of Mo_2_S_3_ at 376 cm^−1^ and 403 cm^−1^, respectively^25^.

**Figure 4:**
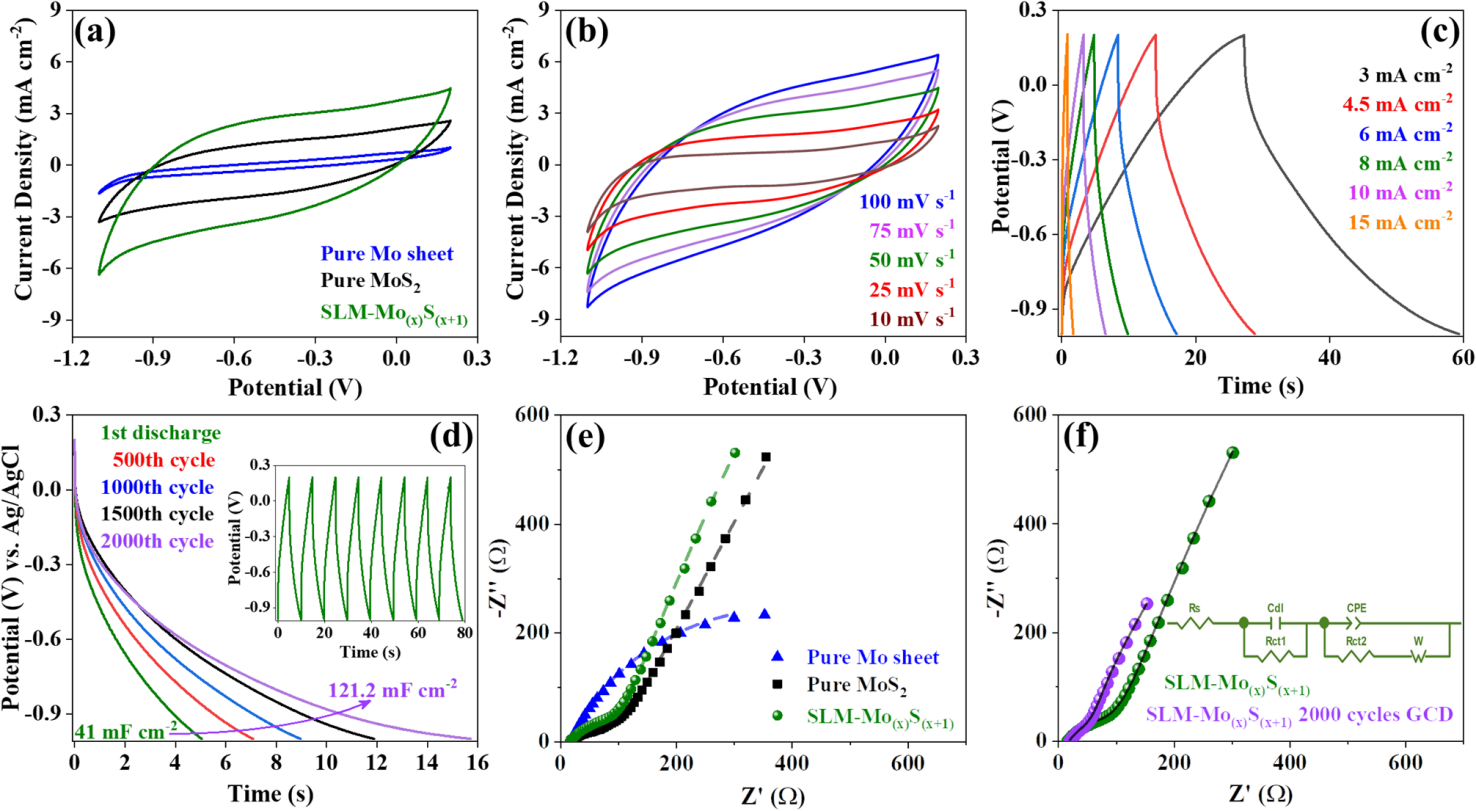
Electrochemical characterization in a 0.5 M Na_2_SO_4_: **(a)** CV curves of pure Mo sheet, pure MoS_2_, and SLM-Mo_(x)_S_(x+1)_ by using a scan rate of 50 mV s^-1^, **(b)** cyclic voltammograms for different scan rates from 10 to 100 mV s^−1^, **(c)** GCD curves for different current densities from 3 to 15 mA cm^−2^, **(d)** discharge retention stability after 500, 1000, 1500, and 2000 cycles of GCD at a current density of 8 mA cm^−2^, and **(e)** Nyquist plot of pure Mo sheet, pure MoS_2_, and SLM-Mo_(x)_S_(x+1)_ in the frequency range between 10^–1^ Hz to 100 kHz, **(f)** Nyquist plot of SLM-Mo_(x)_S_(x+1)_ in the frequency range between 10^–1^ Hz to 100 kHz before and after 2000 cycles of GCD at a current density of 8 mA cm^−2^.

Cyclic voltammograms (CV) show considerably enhanced current densities for the SLM-Mo_(x)_S_(x+1)_ sample compared to both pure Mo and MoS_2_ electrodes (Fig. 4a). Due to the presence of the Mo_2_S_3_ nanoparticles as well as the laser-based thinning and crystallographic phase transformation of MoS_2_ from 2H to 1T, it is evident that the CV loops retained their nearly rectangular shapes even at high scan rates (100 mV s^−1^) (Fig. 4b)^7^. Quasi-symmetrical triangle plots of the galvanostatic charge–discharge (GCD) at different current densities revealed nearly 1.0 V in output voltage even at a current density of 15 mA cm^−2^ for the SLM- Mo_(x)_S_(x+1)_ electrodes (Fig. 4c). Herein, we observed considerable capacitance enhancement up to 2000 cycles of GCD at a current density of 8 mA cm^−2^. This confirms an increased areal capacitance (from 41 to 121.2 mF cm^−2^) and a decreased iR voltage drop. The enhanced capacitance induced by increasing the number of GCD cycles can be attributed to facilitated intercalation of Na^+^ cations, activation of the electrode texture, and improved electrical conductivity (Fig. 4d). It can also be ascribed to the penetration of electrolyte into micro-holes of the structure, successively activating deeper electro-active sites, and possibly a continued activation of sub-layers beneath the SLM-Mo_(x)_S_(x+1)_ surface^36,37^.

As shown in Fig. 4e, faster kinetic of the charge transfer at the interface offered by the layered 1T-MoS_2_, separated by Mo_2_S_3_ nanoparticles, resulted in a considerably lower R_ct_ (75.36 Ω) and a quasi-semicircle shape of the Nyquist plot of SLM-Mo_(x)_S_(x+1)_ compared to pure Mo. The higher slope of the diffusion impedance in the low-frequency region for SLM-Mo_(x)_S_(x+1)_ compared to pure 2H-MoS_2_, confirms facilitated diffusion and accumulation of Na^+^ charges into the defect-rich 1T-MoS_2_ nanolayers within the porous surface (Fig. 4e)^38^. Comparing the SLM-Mo_(x)_S_(x+1)_ electrode before and after 2000 cycles GCD in Fig. 4f, showed a considerably decreased diameter of the quasi-semicircle of the Nyquist plot (charge transfer resistance) at high frequencies as well as an increased slope of the diffusion impedance in the low-frequency region. This further verify faster kinetics of the interface charge transfer and facilitated Na^+^ intercalation into the deeper active sites and spaces of electrode materials, respectively, by increasing the number of GCD cycles.

**Figure 5:**
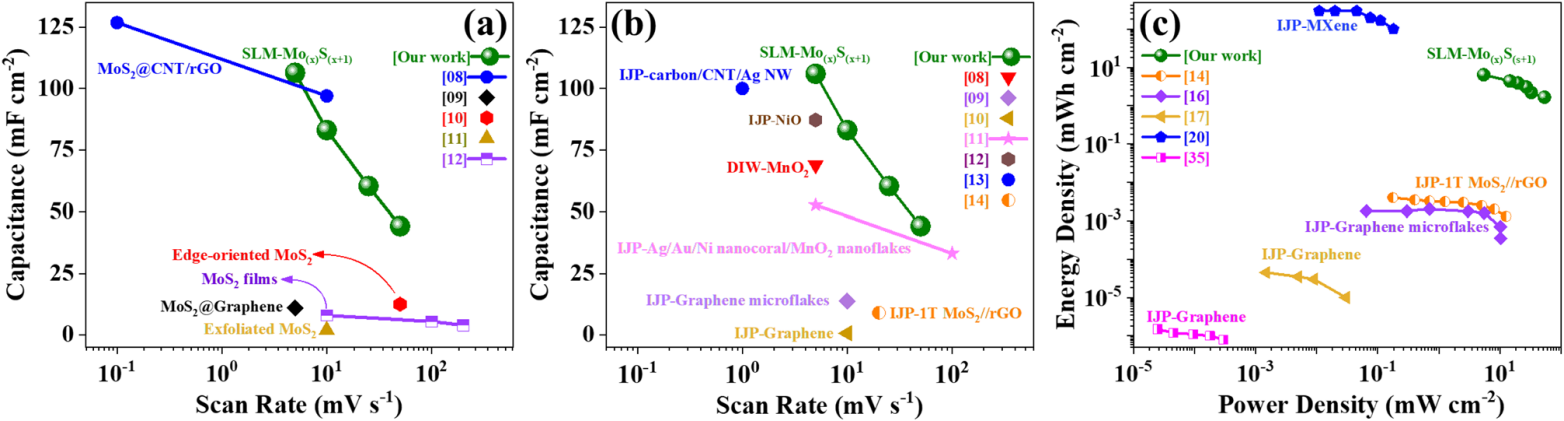
**(a)** Areal rate capacitance for different MoS_2_-based structures processed by various conventional methods for energy storage devices (the weight percentage in parenthesis indicates the amount of MoS_2_ in the structure); **(b)** areal rate capacitance for different materials processed with various 3D printing methods for energy storage applications and **(c)** Ragone plot comparing the areal power density versus areal energy density of 3D-printed electrodes intended for energy storage applications.

Areal rate capability studies of the SLM-Mo_(x)_S_(1+x)_ electrode revealed capacitance retention of 53% even at a scan rate of 50 mV s^−1^ (44.16 mF cm^−2^) compared to a low scan rate of 10 mV s^−1^ (83.1 mF cm^−2^). This is considerably higher than their counterpart electrodes processed by different 3D printing techniques (Fig. 5a,b). Furthermore, according to the Ragone plot (Fig. 5c), the SLM-Mo_(x)_S_(x+1)_ electrodes offered higher power (53.34 mW cm^−2^) and energy density (1.66 mWh cm^−2^) compared with ink-jet printed MXene-^39^, and graphene-based electrodes^16,17,40^, as well as 1T-MoS_2_/graphene-based electrodes^14^.

## Concluding remarks

The successful fabrication of SLM-processed MoS_2_/Mo_2_S_3_ electrodes with a remarkable capacitive behavior compared to other 3D-printed counterparts provides a new possibility to manufacture the next generation of 3D-printed electrochemical devices. In this study, we elucidate that a high-energy Nd:YAG laser can simultaneously exfoliate and tune the structure of MoS_2_, distribute electroactive Mo_2_S_3_ nanoparticles into the structure of the nanocomposite, and therefore considerably enhance the power density and energy density of 3D-printed structures. The preliminary results promote further research on laser-based processing of 2D nanomaterials for a wide range of functional structures e.g., EECSS, high-temperature solid-state energy conversion/storage systems, aerospace parts, and green energy storage device fabrication, even in space.

## Materials and methods

**Figure 6:**
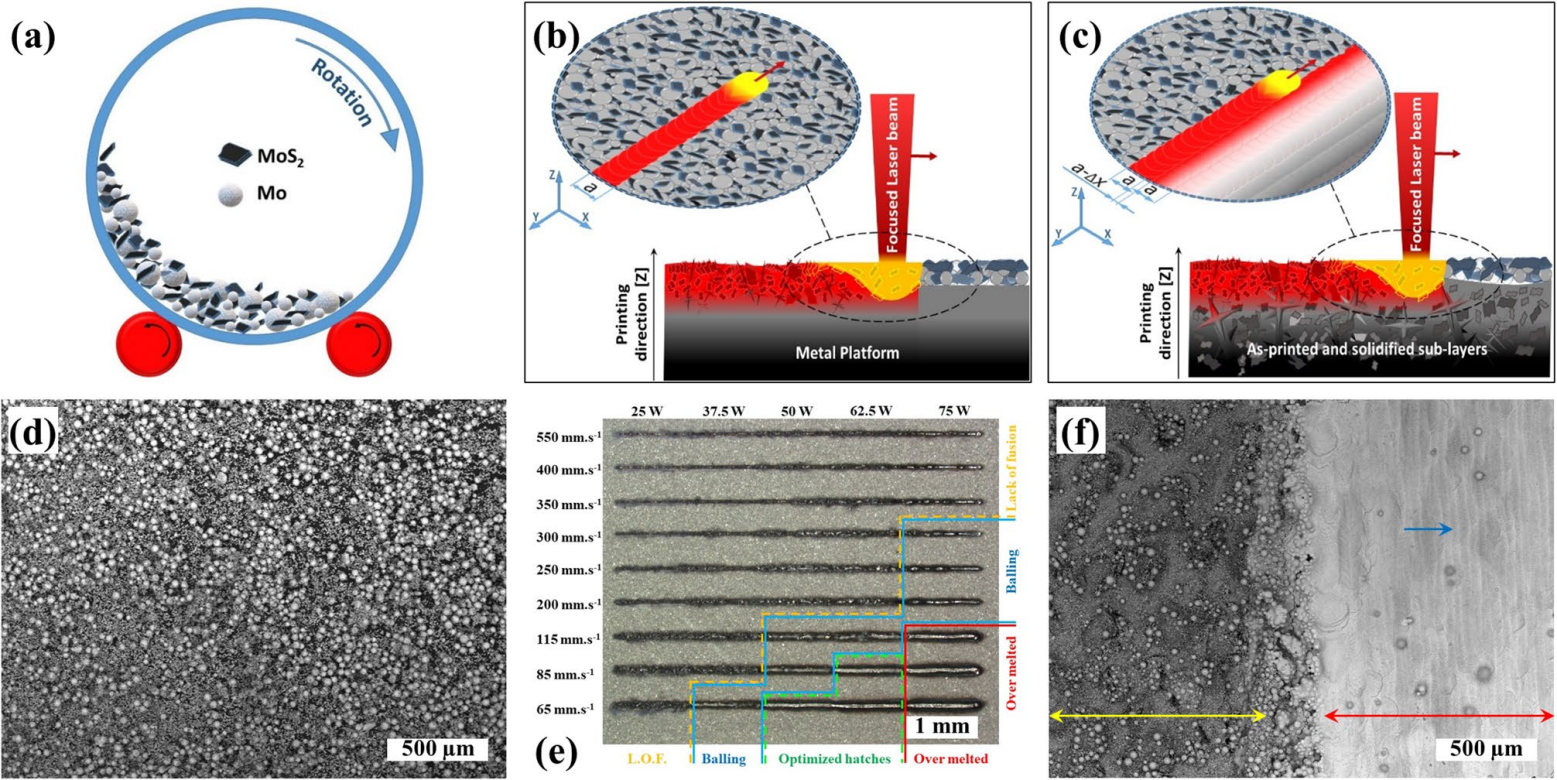
Schematic illustrating the **(a)** mixing of Mo and MoS_2_ powders, **(b)** single laser scanning, and **(c)** laser-based fusion of the powder bed with partial remelting of the previously solidified layer. **(d)** SEM images of a homogenous mixture of Mo and MoS_2_ powders, **(e)** stereomicrograph of laser single scan as a function of energy density, and **(f)** SEM image of the half-melted sample showing the boundary between the melted/solidified (red arrow), un-melted powder bed (yellow arrow), and hatch overlap during continues single laser scanning of powder bed (blue arrow).

Gas atomized powder (GAP) of commercial pure molybdenum (Mo, 99.99%) was used as the feedstock to fabricate both the current collector and the electroactive nanocomposite. Pure molybdenum disulfide powder (2H-MoS_2_, 99.99%, Sigma Aldrich) was used as feedstock for SLM processing of the electroactive nanocomposite. A mixture of Mo and MoS_2_ (20 wt.%) was prepared by mixing for 6 h at 20 rpm to obtain a homogenous mixture (Fig. 6a,d). The Na_2_SO_4_ (≥ 99.0%, Merck) and ultrapure water, with a conductivity of 18.2 MΩcm and a pH of 6.5 ± 0.1 at 25 °C, was used to prepare the 0.5 M Na_2_SO_4_ (pH 5.5 ± 0.1) electrolyte for the electrochemical investigations.

A laser scattering particle size distribution analyzer (LPSA, LA-950, HORIBA, Japan) was used to evaluate the particle size distribution of the Mo and MoS_2_ powders. A Realizer GmbH SLM-50 device equipped with Nd:YAG laser was used for SLM processing of pure Mo as a substrate/current collector and MoS_2_/Mo_2_S_3_ nanocomposite as the electroactive structure. The 316L substrate platform was used to fabricate the samples at ambient conditions. Laser single scanning (LSS) was carried out as a function of energy density to obtain the optimized printing parameters, where the energy density is defined in Eq. (1):

1 $${E}_{d}=P/(v.h.t)$$

where $P$ is the laser power, $v$ is the laser scan speed, $h$ is the hatch distance, and $t$ is the layer thickness. The hatch distance and layer thickness were kept constant at 0.045 mm and 0.025 mm, respectively. The schematic of single laser scanning on a powder bed to form a single hatch has been illustrated in Fig. 6b. commensurately shown in Fig. 6e, the laser power and scan speed were varied between 25 and 75 W and 65–550 mm s^−1^, respectively. The disk-shaped electrodes were fabricated of a mixture of Mo and MoS_2_ (20 wt.%) by using optimized SLM parameters (laser power − 62.5 W and scan speed − 85 mm s^−1^). Figure 6c illustrates the continued scanning of powder-bed leading to consolidation and formation of one layer onto the previously printed and solidified sub-layers (Supplementary Information). Each layer can contain approximately 14 mg MoS_2_. Shown in Fig. 6f, both the sintered particles in the boundary between melted/solidified region (red arrow) and un-melted powder bed (yellow arrow) and overlapping of hatches (vertical lines indicated by the blue arrow) are evident. Hatch size (a) and hatch overlap (a − ∆x) have been illustrated in Fig. 6b,c, respectively.

A stereomicroscope (V20, Zeiss, Germany) was used to study the effect of different single laser scanning parameters on the stability of the melt pool to optimize the 3D printing parameters (Fig. 6e). An ultrasonic bath (USC300TH, VWR, Malaysia) was used to prepare and degas the 0.5 M Na_2_SO_4_ electrolyte as well as to clean the surface of the 3D-printed electrodes. A standard pH meter (PHM210, Radiometer Analytical-Hack, France) was used to measure the pH of the Na_2_SO_4_ electrolyte. A PARSTAT MC multichannel potentiated (AMETEK, Princeton Applied Research, USA), an Ag/AgCl reference electrode (AMETEK, Princeton Applied Research, USA), and a Pt wire counter electrode (CHI115, CH Instruments Inc., USA) were used for the electrochemical studies. The surface area of the working electrode (anode) was 0.33 cm^2^. Cyclic voltammetry (CV) was carried out using scan rates between 10 and 100 mV s^−1^ in a potential window between −1.1 and 0.2 V. Electrochemical potentiostatic impedance (EIS) measurements were carried out in the frequency range between 10^–1^ Hz and 100 kHz (Figure S4b). Galvanostatic charge–discharge (GCD) measurements were carried out at different current densities (3, 4.5, 6, 8, 10, and 15 mA cm^−2^). 2000 cycles of GCD at a current density of 8 mA cm^−2^ were performed on the electrodes to evaluate their stability and capacity retention.

X-ray diffraction (XRD) studies were carried out by using a Smartlab (Rigaku, Japan) diffractometer equipped with a rotating 9 kW Cu anode X-ray tube with Cu-Kα1 radiation (λ = 1.5406 Å), and a silicon strip detector D/teX Ultra. Based on the Rigaku database, ICDD card numbers 9008543, 1531960, and 1531960 were selected to identify the XRD patterns of Mo, MoS_2_, and Mo_2_S_3_, respectively. Raman measurements were carried out using a Horiba LabRAM HR800 micro-Raman system equipped with a cooled multichannel CCD detection system in the back-scattering configuration with a spectral resolution of 1 cm^−1^. A Nd:YAG laser (λ = 532 nm) was used for excitation with a laser spot size diameter of approximately 5 μm. A high-resolution scanning electron microscope (HR-SEM, Zeiss MERLIN, Germany) was employed to study the surface morphology of 3D-printed samples in both back-scattered (BE) and secondary (SE) electron modes (Suplementary Information).

## Supplementary Information

Supplementary Video 1.

Supplementary Information.

## Data Availability

All data generated or analyzed during this study are included in this published article, and the datasets used and analyzed during the current study are available from the corresponding author on reasonable request.
