# Supplementary figures and images for: SLM-processed MoS2/Mo2S3 nanocomposite for energy conversion/storage applications
Source: Sci Rep. 2022 Mar 23;12:5030. doi: 10.1038/s41598-022-08921-7 (PMC8943036; doi:10.1038/s41598-022-08921-7)

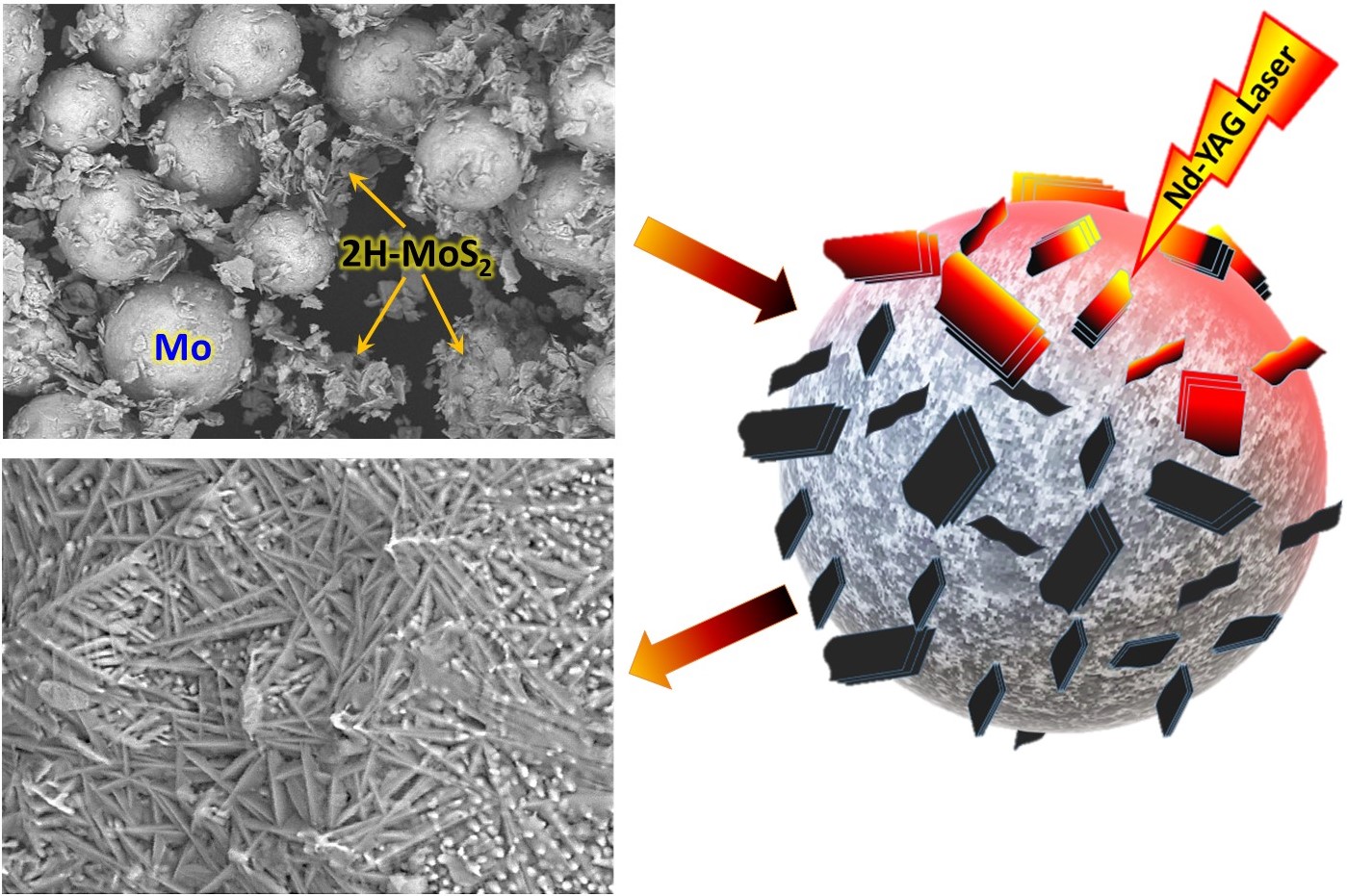

Supplement: Supplementary file 2 — Supplementary Information. [file 41598_2022_8921_MOESM2_ESM.jpg]
